# The value of primary care networks for molecular surveillance of paediatric respiratory infections

**DOI:** 10.1007/s10096-025-05353-9

**Published:** 2025-11-29

**Authors:** Cristina Andrés, Anna Creus-Costa, Aida Perramon-Malavez, Jorgina Vila, Patricia Nadal-Barón, Anna Gatell, Ramona Martín-Martín, Eduard Fernández, Marisa Ridao, Mireia Biosca, Almudena Sánchez, Olga Salvadó, Maria Chiné, Lidia Sanz, Dolors Canadell, Esperança Macià, Mònica Vilà, Gloria Ruiz, Clara Prats, Andrés Antón Pagarolas, Antoni Soriano-Arandes

**Affiliations:** 1 Respiratory Viruses Unit, Virology Section, Microbiology Department, Vall d’Hebron Hospital Universitari, Vall d’Hebron Institut de Recerca (VHIR), Vall d’Hebron Barcelona Hospital Campus, Passeig Vall d’Hebron 119-129, Barcelona, 08035 Spain; 2 Pediatric Hospitalization Unit, Hospital Infantil i de la Dona Vall d’Hebron, Vall d’Hebron Barcelona Hospital Campus, Barcelona, Catalonia Spain; 3 Infection and Immunity in Pediatric Patients Research Group, Vall d’Hebron Institut de Recerca (VHIR), Barcelona, Catalonia Spain; 4 Primary Care Services Information System (SISAP), Institut Catala de la Salut, Barcelona, Spain; 5 Equip Territorial Pediatria Garraf, Barcelona, 08800 Spain; 6 CAP Marià Fortuny, Reus, Tarragona, 43205 Spain; 7 EAP Vic Nord, Vic, Barcelona, 08500 Spain; 8 EAP Sant Vicenç dels Horts, Barcelona, 08620 Spain; 9 EAP Les Borges Blanques, Lleida, 25400 Spain; 10 CAP Les Hortes, Barcelona, 08004 Spain; 11 CAP Llibertat, Reus, Tarragona, 43201 Spain; 12 CAP Almacelles, Lleida, 25100 Spain; 13 CAP Seròs, Lleida, 25183 Spain; 14 CAP Barberà del Vallès, Barcelona, 08210 Spain; 15 CAP Manlleu, Barcelona, 08560 Spain; 16 EAP Horta, Barcelona, 08032 Spain; 17 Pediatria Dels Pirineus SCCLP, Lleida, Catalonia Spain; 18 Department of Physics, Institute for Research and Innovation in Health (IRIS), Universitat Politècnica de Catalunya (UPC-BarcelonaTech), Barcelona, Spain; 19 Centro de Investigación Biomédica en red de Enfermedades Infecciosas CIBERINFEC, Instituto Carlos III, Madrid, Spain; 20 Department of Pediatrics, Serveis de Salut Integrats del Baix Empordà, Palamós, Girona Spain

**Keywords:** Paediatric population, Respiratory infection, Primary care, Co-infections

## Abstract

**Background:**

This study aimed to provide a comprehensive overview of SARS-CoV-2 and other respiratory viruses co-infections and analyse the value of Primary Care Centres (PCCs) as a sentinel network for molecular surveillance of paediatric respiratory viral infections in Catalonia (Spain).

**Methods:**

Between October 2021 and April 2024, upper respiratory tract samples were collected from children under 15 years of age presenting with acute respiratory symptoms at different PCCs across Catalonia. The detection of respiratory viruses was performed using commercial multiplex RT-PCR and transcription-mediated amplification-based assays. The genetic characterisation of select viruses (adenoviruses (AdV), enteroviruses (EV), influenza viruses, SARS-CoV-2) was performed via partial or whole genome sequencing. The results were then compared with hospital-based data and the regional surveillance system (SIVIC).

**Results:**

Among 1,401 positive samples from 1,329 cases, the most prevalent viruses were rhinovirus (RV) (22.77%), SARS-CoV-2 (12.35%), influenza A(H3) viruses (11.06%) and AdV (9.21%). Viral circulation followed typical seasonal patterns, with RV and AdV detected year-round, and influenza and respiratory syncytial virus peaking in winter, showing prevalences similar to those observed in hospital settings and broader community settings. Co-infections were frequent (up to 53.3% for bocavirus), while influenza and SARS-CoV-2 showed the lowest co-infection rates, suggesting possible viral interference. Genomic analysis revealed circulation of different EV (e.g., EV-D68, CV-A6, E-11, etc.) and AdV (B3, C2) types, multiple FLUAV and FLUBV genetic clades and SARS-CoV-2 variants consistent with national waves.

**Conclusions:**

This study highlights the complexity of respiratory virus circulation and co-infections dynamics in paediatric primary care patients. A notable observation was the generally similar viral distribution between PCCs and the community, reinforcing the value of studying this population. The findings also underscore the importance of continued molecular surveillance to inform public health strategies and clinical management of respiratory infections in children.

**Supplementary Information:**

The online version contains supplementary material available at 10.1007/s10096-025-05353-9.

## Introduction

Respiratory viral infections are a significant cause of morbidity in paediatric populations worldwide, ranging from asymptomatic or mild upper respiratory tract infections to severe lower respiratory tract diseases, including pneumonia and bronchiolitis, particularly in infants and young children. The viruses most commonly associated with these infections include human respiratory syncytial virus (HRSV), influenza A (FLUAV) and B (FLUBV) viruses, adenoviruses (AdV), enteroviruses (EV) and, more recently, SARS-CoV-2 [1, 2]. These infections place a considerable burden on healthcare systems, leading to increased hospitalisation rates, inappropriate antibiotic use and a heightened risk of secondary bacterial infections [3–5].

Co-infection with multiple respiratory viruses is an increasingly recognised but still underestimated concern in paediatric patients despite the growing use of multiplex molecular methods for comprehensive detection of respiratory viruses. Viral co-infections have been associated with variable clinical outcomes, sometimes exacerbating disease severity, while in other cases viral interference mechanisms may suppress certain pathogens [6]. The SARS-CoV-2 pandemic has further complicated this landscape by altering transmission dynamics and interactions between respiratory pathogens [7]. Understanding the prevalence, molecular characteristics and co-infection dynamics of respiratory viruses in children is essential to guide clinical management and public health strategies. This study aimed to provide a comprehensive overview of SARS-CoV-2 and other respiratory viruses co-infections and analyse the value of Primary Care Centres (PCCs) as a sentinel network for molecular surveillance of paediatric respiratory viral infections in Catalonia (Spain).

##  Material and methods

### Patients and samples

**Fig. 1:**
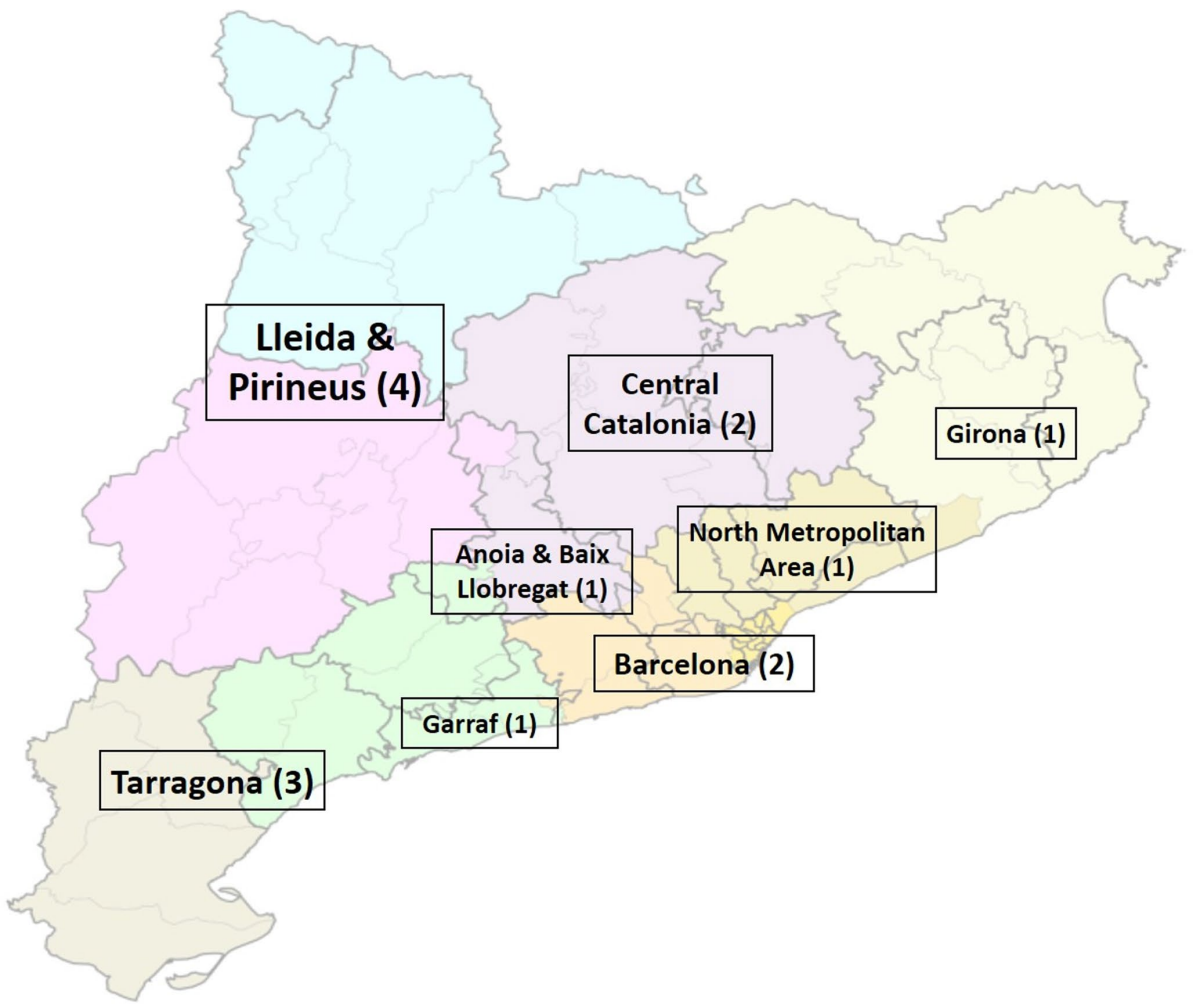
Distribution of participating primary care centres over Catalonia (number of centres per region)

From October 2021 (week 40/2021) to April 2024 (week 17/2024), upper respiratory tract specimens (naso/oropharyngeal swabs) were collected for laboratory-confirmation of respiratory viruses (FLUAV, differentiating H1pdm09 and H3 subtypes, FLUBV, HRSV-A and B, AdV, EV, Human Metapneumovirus [HMPV], Parainfluenza viruses [hPIV-1, hPIV-2, hPIV-3, and hPIV-4], Bocavirus [BoV], human coronaviruses [CoV] 229E, NL63 and OC43, rhinovirus [RV], and SARS-CoV-2) from paediatric patients (< 15 years old) with suspected acute respiratory tract infection (ARTI; defined as the presence of two or more signs or symptoms such as fever, cough, runny nose or nasal congestion, or sore throat) (https://www.cdc.gov/flu/glossary/index.html) who were attended at several primary care centres distributed over Catalonia (Fig. 1). Demographic features (sex and age) were collected from all cases. Moreover, laboratory-confirmation from respiratory specimens obtained from patients attended at the reference hospital (Hospital Vall d’Hebron) during the same period were also included in this study, in addition to data from the Information System for Infection Surveillance in Catalonia (SIVIC; https://sivic.salut.gencat.cat/). This approach enabled the assessment of hospital- and primary care-based populations with our cohort for statistical analysis by the comparison of two proportions and chi-squared test for comparison between age groups. The statistical significance level was set at a p-value < 0.05 [8].

### Laboratory-confirmation of SARS-CoV-2 and other respiratory viruses

Detection of SARS-CoV-2 was performed using high-throughput, automated transcription-mediated amplification based-assays (Aptima SARS-CoV-2, Hologic Inc., USA) on the Panther platform. Other viruses were detected using commercial real-time multiplex RT-PCR (Allplex Respiratory Panels 1 A-3, Seegene, Korea) on the Microlab STARlet system (Hamilton, USA) [9].

### Molecular characterisation of respiratory viruses by sequencing

Genetic characterisation was performed by partial (EV and AdV) or complete (FLUAV, FLUBV, and SARS-CoV-2) genome sequencing. EV typing was performed by the phylogenetic analyses of the partial viral protein 1 (VP1) coding-region following the protocol recommended by the World Health Organisation (WHO) [10], with minor modifications [9]. For AdV, the partial *hexon* gene (conserved and L1 regions) was sequenced [11, 12], and the AdV typing was subsequently performer through phylogenetic analysis of both regions. Whole-genome sequencing (WGS) was performed following the ARTIC v4.1 or v5.3.2 protocols (https://artic.network/ncov-2019) using the COVIDseq test (Illumina, USA) for SARS-CoV-2 and primers described by Zhou et al. 2009 for FLUAV [13, 14] and FLUBV [13, 14]. Bioinformatic analysis was performed using an *in-house pipeline* to obtain consensus sequences of SARS-CoV-2 and influenza viruses for genetic characterisation.

## Results

During the study period, a total of 1,462 samples (Table 1) from 1,329 cases were collected from the PCCs (53% were male and 54.5% <5 years old), of which 1,401 (96%) resulted positive for one or more viruses. With regard to the comorbidities and the vaccination status of the patients studied, information was available for 986 patients (74.2%). The most prevalent comorbidities were asthma (58 cases, 5.9%), obesity (16 cases, 1.6%), prematurity (15 cases, 1.5%) and chronic neurological disease (11 cases, 1.1%). A few cases had congenital heart disease (7, 0.7%), chronic lung disease (3, 0.3%), chronic kidney disease (3, 0.3%), coeliac disease (2, 0.2%), HIV infection (1, 0.1%), chronic neurological disease (1, 0.1%), and oncohaematological disease (1, 0.1%). Regarding vaccination, 212 (21.5%) of patients had received COVID-19 vaccine once, 88 (8.9%) influenza vaccine, and the majority were up to date with their vaccination schedule (97.8%, 964).

**Table 1:** Global distribution of viral laboratory-confirmed cases between hospital, primary care (PCC) settings and SIVIC during the study period. Statistics for proportions are also shown between PCCs and Hospital/SIVIC data. P-values statistically significant are indicated with an asterisk. Shown in italics those viral detections mostly observed

	Hospital (*N*)	%	PCC vs. Hospital	PCCs (*N*)	%	PCC vs. SIVIC	SIVIC	%
FLUAV(H1)pdm09	1886	2.15%	*p* = 0.35	56	4.00%	*p* = 0.63	1179	5.51%
*FLUAV(H3)*	*2749*	*3.13%*	p < 0.0001*	*155*	*11.06%*	*p = 0.17*	*1696*	*7.92%*
FLUBV	766	0.87%	*p*** = **0.004*	68	4.85%	*p* = 0.96	1013	4.73%
HRSV-A	1264	1.44%	*p*** =** 0.002*	84	6.00%	*p* = 0.07	507	2.37%
HRSV-B	1521	1.73%	*p* = 0.06	68	4.85%	*p* = 0.41	641	2.99%
HMPV	1333	1.52%	*p*** =** 0.008*	78	5.57%	*p* = 0.74	1389	6.49%
AdV	1394	1.59%	*p*** < **0.0001*	129	9.21%	*p*** =** 0.001*	654	3.05%
CoV-229E	241	0.27%	*p* = 0.99	4	0.29%	*p* = 0.85	290	1.35%
CoV-NL63	204	0.23%	*p* = 0.81	9	0.64%	*p* = 0.86	279	1.30%
CoV-OC43	708	0.81%	*p* = 0.15	42	3.00%	*p* = 0.82	789	3.68%
*RV*	*6627*	*7.55%*	p < 0.0001*	*319*	*22.77%*	*p = 0.02*	*6159*	*28.76%*
BoV	750	0.85%	*p* = 0.43	31	2.21%	*p* = 0.53	200	0.93%
hPIV-1	158	0.18%	*p* = 0.55	14	1.00%	*p* = 0.96	184	0.86%
hPIV-2	196	0.22%	*p* = 0.47	17	1.21%	*p* = 0.97	240	1.12%
hPIV-3	921	1.05%	*p* = 0.08	52	3.71%	*p* = 0.85	910	4.25%
hPIV-4	420	0.48%	*p* = 0.50	22	1.57%	*p* = 0.96	328	1.43%
EV	1415	1.61%	*p*** =** 0.008*	80	5.71%	*p* = 0.15	583	2.72%
*SARS-CoV-2*	*20,719*	*23.61%*	p = 0.0005*	*173*	*12.35%*	p = 0.009*	*4374*	*20.42%*
TOTAL	87,742			1401			21,415	

### Respiratory viruses circulation and co-infections

Overall, RV was the most detected virus (319, 22.77%), followed by SARS-CoV-2 (173, 12.35%), influenza A(H3) virus (155, 11.06%), AdV (129, 9.21%). These results were consistent with those observed at hospital settings and a broader community surveillance system during the same period (Table 1). Similar prevalences for most viruses detected between PCCs and SIVIC were noticed, denoting a comparable viral detection pattern across different settings, except for AdV, Influenza A, Influenza B, HRSV-A, HMPV and EV more frequently observed in PCCs than in hospital, while RV and SARS-CoV-2 in SIVIC and hospital, respectively. It is worth to mention that those viruses related to mild infections were more representative among PCCs and SIVIC, as expected. As demonstrated in Table 2 and Supplementary Fig. 1, the distribution of viral detections also varied according to age group. For instance, RV, AdV and hPIVs exhibit equal distribution across all age groups. However, certain age groups, such as those under two years and five to 14 years, were more indicative of specific pathogens, HRSV and influenza, respectively.

**Table 2:** Distribution by age group of viral detections observed during the study period

	AGE GROUP				
VIRUS	< 2	2–4	5–14	TOTAL	*p*-valor
FLUAV	25	53	131	**209**	**< 0.0001**
FLUBV	10	8	50	**68**	**< 0.0001**
HRSV	98	40	14	**152**	**< 0.0001**
HMPV	23	27	28	**78**	0.232
AdV	45	37	47	**129**	0.855
CoVs	22	20	13	**55**	0.264
RV	125	85	108	**318**	0.200
BoV	19	9	3	**31**	**0.0018**
hPIVs	45	29	31	**105**	0.09
EV	21	43	16	**80**	**< 0.0001**
SARS-CoV-2	64	21	87	**172**	**< 0.0001**
TOTAL	**497**	**372**	**528**	1397*	

*4 detections were not included that corresponded to 15–17 years to perform the statistical analyses

**Fig. 2:**
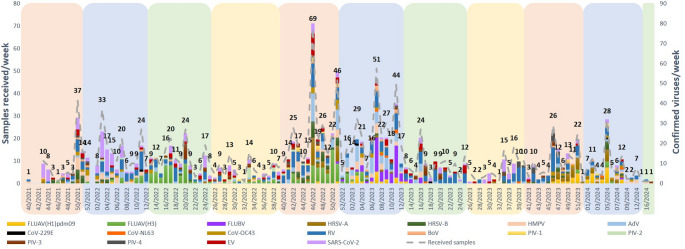
Weekly distribution of viral detections from samples received during the study period. Annual seasons are represented in colours: , ,  and

**Fig. 3:**
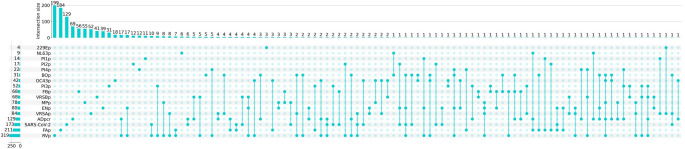
Coinfections observed between different viruses during the study period

Additionally, the distribution and circulation of respiratory viruses varied according to the time period, following expected seasonality: RV or AdV were detected year-round, while influenza, HRSV or SARS-CoV-2, were more prevalent in winter months, and EV, was mostly detected during autumn and spring months (Fig. 2). Moreover, the observed mono- (842, 75%) and co-infections for one or more viruses (284, 25%) are depicted in Fig. 3 and showed that influenza A viruses (6.5%) and SARS-CoV-2 (12.4%) exhibited the lowest rates of co-infection with other respiratory viruses. In contrast, BoV (53.3%), CoV-OC43 (33.3%) and EV (22.5%) were frequently detected alongside other pathogens.

### Genetic characterisation of respiratory viruses

Regarding the molecular characterisation of specific viruses, sequencing data of AdV-positive samples revealed the predominance of species B (B3; 50/94, 53%) and C (C2; 22/94, 23%) (Supplementary Fig. 2), both typically associated with respiratory infections [15]. For EV, the genetic analysis confirmed the presence of up to 18 distinct types within EV-A, -B and -D species, including EV-D68, echovirus 18 (E-18), and echovirus 11 (E-11), as the most observed types (Supplementary Fig. 3).

On the other hand, the distribution of SARS-CoV-2 lineages (122 cases) in paediatric cases was representative of the broader community transmission patterns perceived during that time, including those observed in hospital settings (Supplementary Fig. 4). The predominant circulating variants change over time, reflecting the dominant sub-lineages during each pandemic wave [13].

**Fig. 4:**
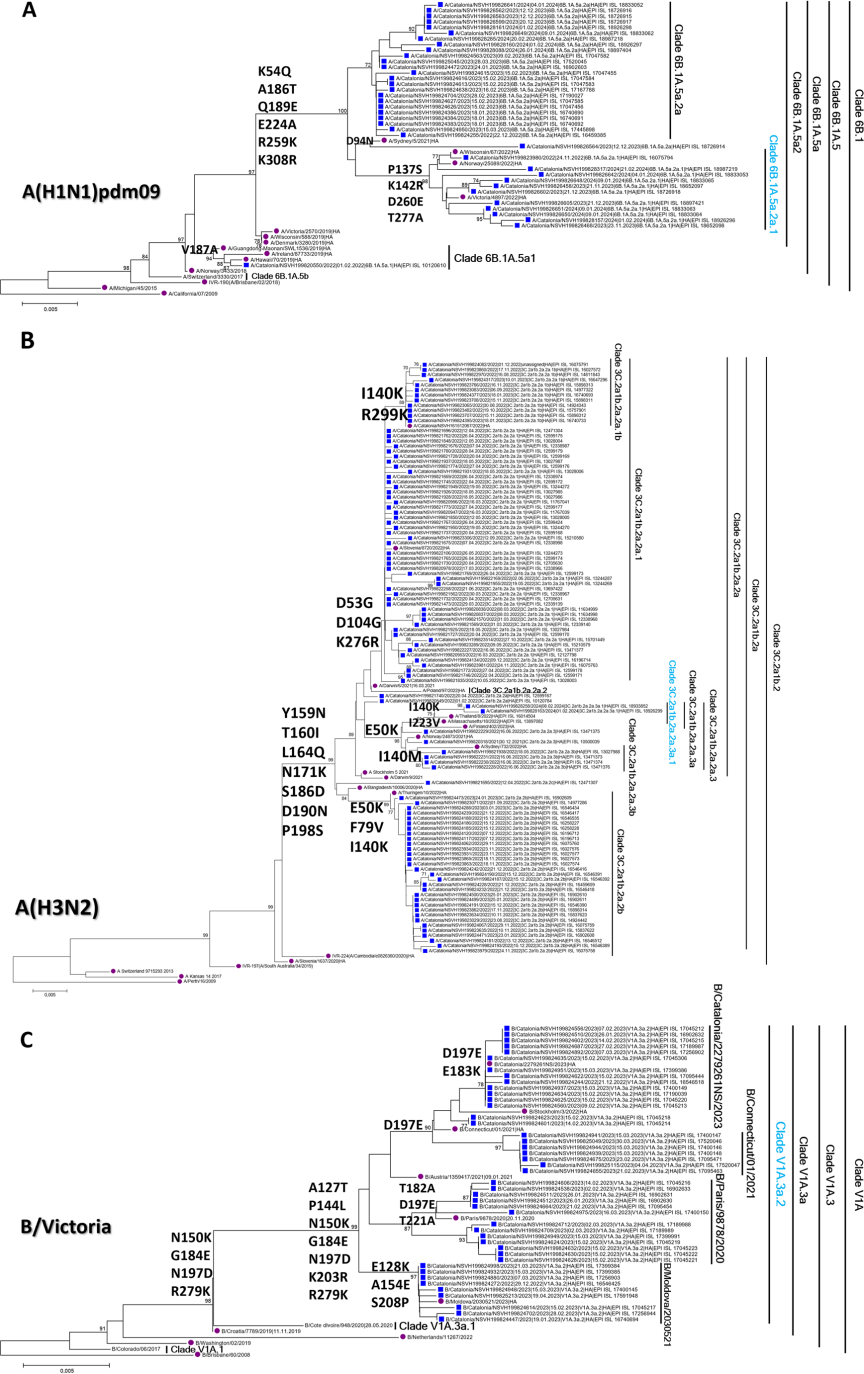
Phylogenies of the domain 1 of the hemagglutinin coding region (HA1) of the influenza A(H1N1)pdm09 (**A**), A(H3N2) (**B**) and B/Victoria viruses (**C**). The samples studied from the PCCs are represented by blue squares, and in purple, the reference sequences used for each subtype/lineage. The genetic clades are described, and the one corresponding to the vaccine strain of the 2023–2024 season (last season studied) is represented in light blue

Regarding other respiratory viruses, a total of 191 samples positive for influenza A (147, 68%) and B (44, 20%) viruses were successfully sequenced, revealing multiple genetic clades with similar antigenic characteristics. Most of them corresponded to vaccine strains circulating in Europe (Fig. 4 and Table 3), in concordance to that observed at hospital settings and SIVIC.

**Table 3:** Influenza genetic clades per type/subtype observed during the study period

Virus	Subtype/Genetic clade	Total
INFLUENZA A	H3N2 - Clade 3 C.2a1b.2a2	107
	H3N2 - Clade 3 C.2a1b.2a.2a.3a1	4
	H1N1pdm09 - Clade 6B.1 A.5a2	16
	H1N1pdm09 - Clade 6B.1 A.5a.2a	10
	H1N1pdm09 - Clade 6B.1 A.5a.2a1	9
	H1N1pdm09 - Clade 6B.1 A.5a.1	1
INFLUENZA B	B/Vic - Clade V1A.3a.2	44
	Not typable	25
**Total**		**216**

## Discussion

This study provides novel insights into the circulation, co-infection dynamics, and genetic diversity of respiratory viruses in paediatric primary care patients in Catalonia, Spain. A notable observation was the generally similar viral distribution between PCCs and the community (SIVIC data) for most of the viruses, highlighting the value of studying the paediatric population for the surveillance of respiratory viruses. Some differences in detection rates for SARS-CoV-2, RV, and AdV were also observed, potentially reflecting variations in population behaviour, healthcare-seeking patterns, or maybe disease severity. For instance, the increased detection of AdV in PCCs may indicate its clinical relevance in symptomatic paediatric cases, while the increased presence of SARS-CoV-2 and RV in community settings likely reflects asymptomatic or mild infections in general population [16, 17].

With regard to the distribution of viral detections across different age groups, research has shown that RV, AdV and hPIVs typically demonstrate broad circulation across age groups, as observed in our series, reflecting recurrent exposures and partial immunity [18, 19]. Conversely, HRSV primarily affects children under the age of two, aligning with the notion of early-life vulnerability and immune immaturity [20]. Concurrently, influenza infections are most prevalent among school-aged children (aged 5–14 years), who function as pivotal amplifiers of community transmission [21]. These findings are consistent with the detections observed in the present cohort and underscore the value and distribution of our series.

In addition, our findings also underscore the seasonal variability of viral prevalence and the complex interactions between co-infecting pathogens. These observations are consistent with previous studies, which suggest that co-infections are relatively common in paediatric patients [22, 23].

### Co-infections and viral interference

The relatively low rates of co-infection between SARS-CoV-2 and influenza viruses observed in our study may be indicative of viral interference mechanisms, as previously described by Gilbert-Girard et al. [23]., whereby the presence of one virus may inhibit the replication or transmission of another within the host. Such effects may occur via competition for cellular entry receptors, modulation of innate immune responses, or induction of antiviral cytokines [23]. These findings warrant further investigation into the underlying mechanisms of such interactions, which could have important implications for understanding viral pathogenesis and optimising paediatric treatment strategies.

### The role of studying paediatric populations

A key strength of this study lies in its focus on paediatric primary care patients, who exhibit distinct epidemiological characteristics compared to adults in the context of respiratory infections. Children, particularly infants, possess immature immune systems, narrower airways at younger ages, and are frequently exposed to novel viral pathogens, making them more vulnerable to infection. As a result, they experience the highest incidence of across all age groups and face an elevated risk of severe disease, mainly due to HRSV infections [24]. In contrast, healthy non-elderly adults typically exhibit the opposite profile, with a more robust and trained immune response capable of recognising pathogens that circulate continuously in the population, even when they appear in evolved forms with minor antigenic changes. This immunological memory confers a certain degree of protection, thereby reducing susceptibility to severe disease. Additionally, higher transmission rates among children contribute significantly to overall viral spread within the population. Children have been often considered vectors of transmission to both household contacts and the wider community [18]. Therefore, enhanced surveillance in this age group is essential for early detection of emerging threats and for guiding prevention and resource allocation strategies within healthcare systems. In this context, our findings support the potential of PCCs as a cost-effective and representative sentinel network for public health surveillance. Integrating PCC-based monitoring within existing national systems has the potential to enhance viral activity monitoring at the community level, improve early warning systems, and bolster preparedness for future respiratory virus epidemics.

### Genetic diversity and surveillance

The observed genetic diversity of respiratory viruses, including influenza, EVs and AdV, reflects ongoing evolution and viral adaptation to host populations, as previously reported [25]. The co-circulation of antigenically drifted strains underscores the need for continuous molecular surveillance. Moreover, the detection of EV types associated with severe disease in other European countries, such as E-11, highlights the importance of sustained monitoring efforts of emerging threats, particularly in paediatric populations at higher risk of severe outcomes [26, 27].

In contrast to studies focusing primarily on adults, which may overlook paediatric-specific viral dynamics, our findings emphasise the value of ongoing molecular surveillance in children. The detection of SARS-CoV-2 variants that mirrored those circulating in the general population suggests that viral transmission dynamics in children are comparable to those in adults, although clinical outcomes may differ significantly due to differences in immune responses. Whole genome sequencing as implemented used in this study, is a powerful tool for tracking viral evolution and understanding transmission patterns, which is critical in the context of evolving respiratory viruses such as SARS-CoV-2 [13].

This study also emphasises the value of targeted paediatric surveillance. Community-wide systems may underreport the burden of illness in children, particularly for viruses presenting with milder symptoms in adults. By focusing on primary care settings, this study provides a more accurate representation of symptomatic viral disease in children. Future research should aim to develop an international collaboration network, including multiple geographical regions and a broader age range, to capture the full spectrum of viral circulation and co-infections across various cohorts.

To conclude, this study demonstrates the dynamic nature of respiratory virus circulation in paediatric primary care patients reflecting patterns similar to those seen in the wider community. The findings reinforce the importance of continued molecular surveillance to guide public health strategies and the clinical management of respiratory infections in children. Furthermore, the identification and classification of respiratory viruses from paediatric primary care settings could serve as a reliable indicator of community-level surveillance of respiratory viruses. This approach could complement or even partially substitute broader surveillance systems in future frameworks.

## Supplementary Information

Below is the link to the electronic supplementary material.

Supplementary Material 1.Percentages of viral detections per age group. With an asterisk, those viruses with statistical significance in Xi^2^ analysis. (PNG 265 KB)

Supplementary Material 2. Weekly distribution of adenovirus (AdV) molecular characterised-cases (A) and total numbers and percentages for each adenovirus genotype (B). (PNG 57.6 KB)

Supplementary Material 3.Weekly distribution of enterovirus molecular characterised-cases (A) and total numbers and percentages for each enterovirus type according to the specie (B). (PNG 152 KB)

Supplementary Material 4.Weekly distribution of SARS-CoV-2 molecular characterised-cases during the study period. (PNG 91.5 KB)

## Data Availability

No datasets were generated or analysed during the current study.
