# Supplementary figures and images for: The value of primary care networks for molecular surveillance of paediatric respiratory infections
Source: Eur J Clin Microbiol Infect Dis. 2025 Nov 29;45(3):845–53. doi: 10.1007/s10096-025-05353-9 (PMC12988900; doi:10.1007/s10096-025-05353-9)

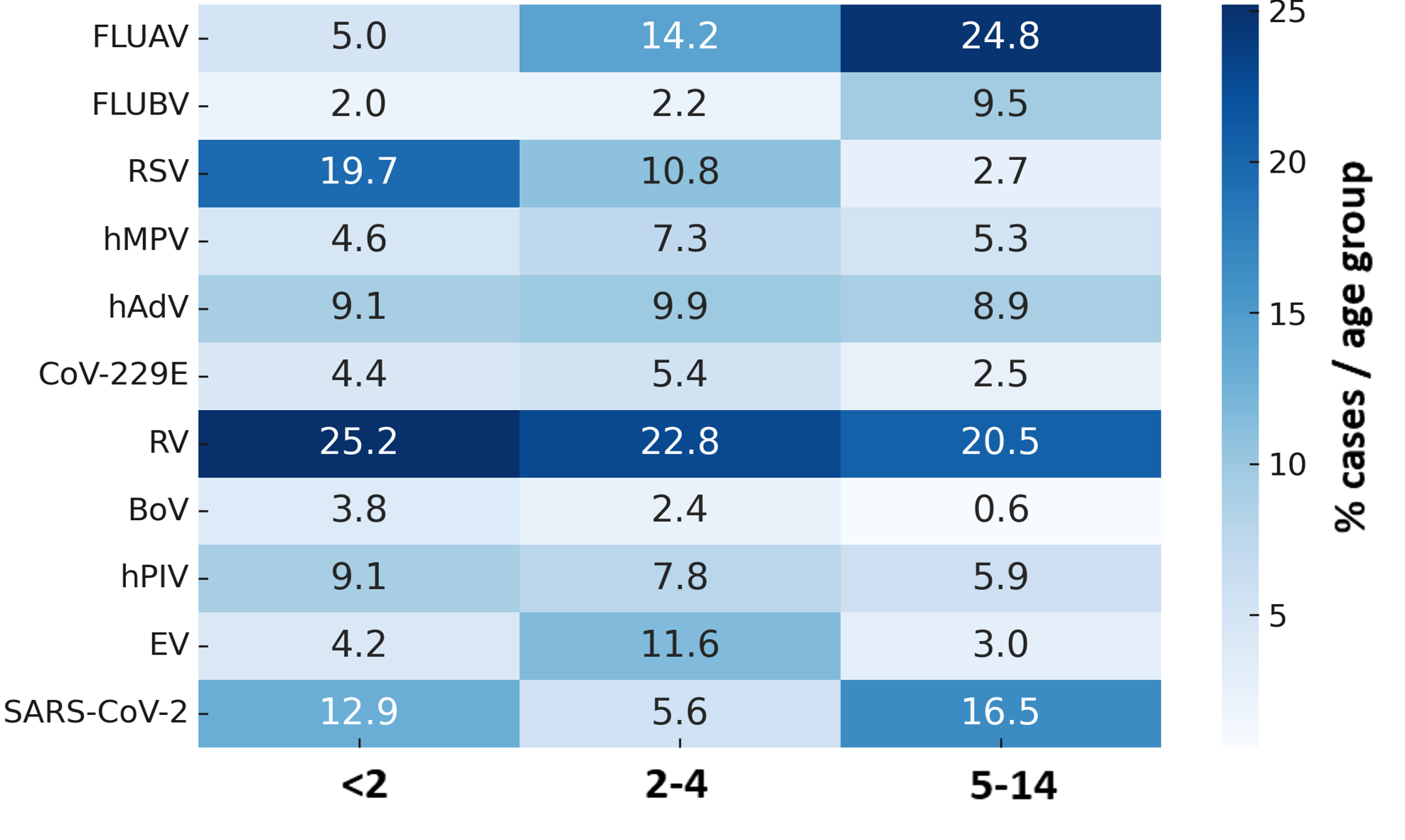

Supplement: Supplementary file 1 — Supplementary Material 1.Percentages of viral detections per age group. With an asterisk, those viruses with statistical significance in Xi2 analysis. (PNG 265 KB) [file 10096_2025_5353_MOESM1_ESM.png]

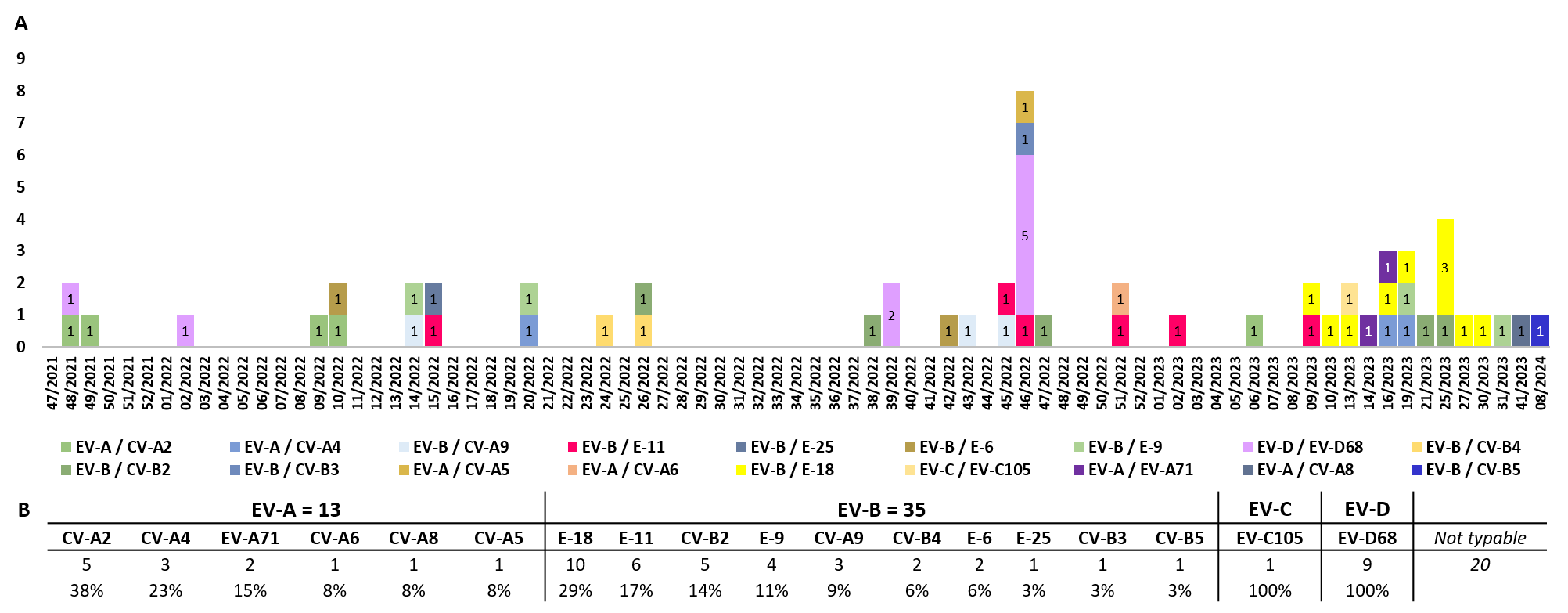

Supplement: Supplementary file 2 — Supplementary Material 2. Weekly distribution of adenovirus (AdV) molecular characterised-cases (A) and total numbers and percentages for each adenovirus genotype (B). (PNG 57.6 KB) [file 10096_2025_5353_MOESM2_ESM.png]

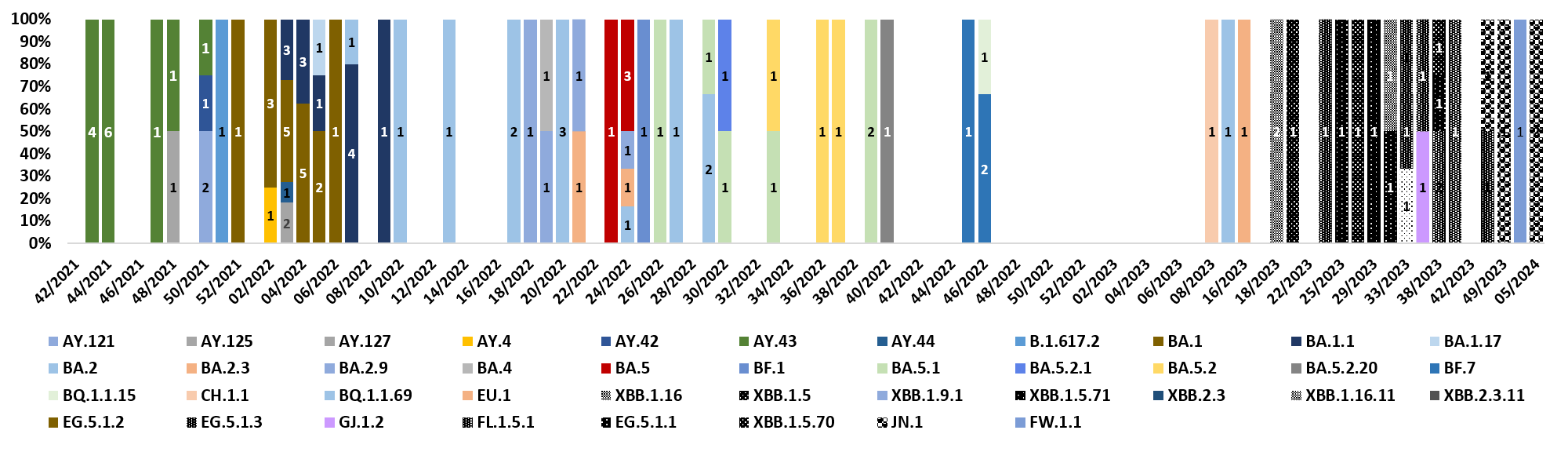

Supplement: Supplementary file 3 — Supplementary Material 3.Weekly distribution of enterovirus molecular characterised-cases (A) and total numbers and percentages for each enterovirus type according to the specie (B). (PNG 152 KB) [file 10096_2025_5353_MOESM3_ESM.png]

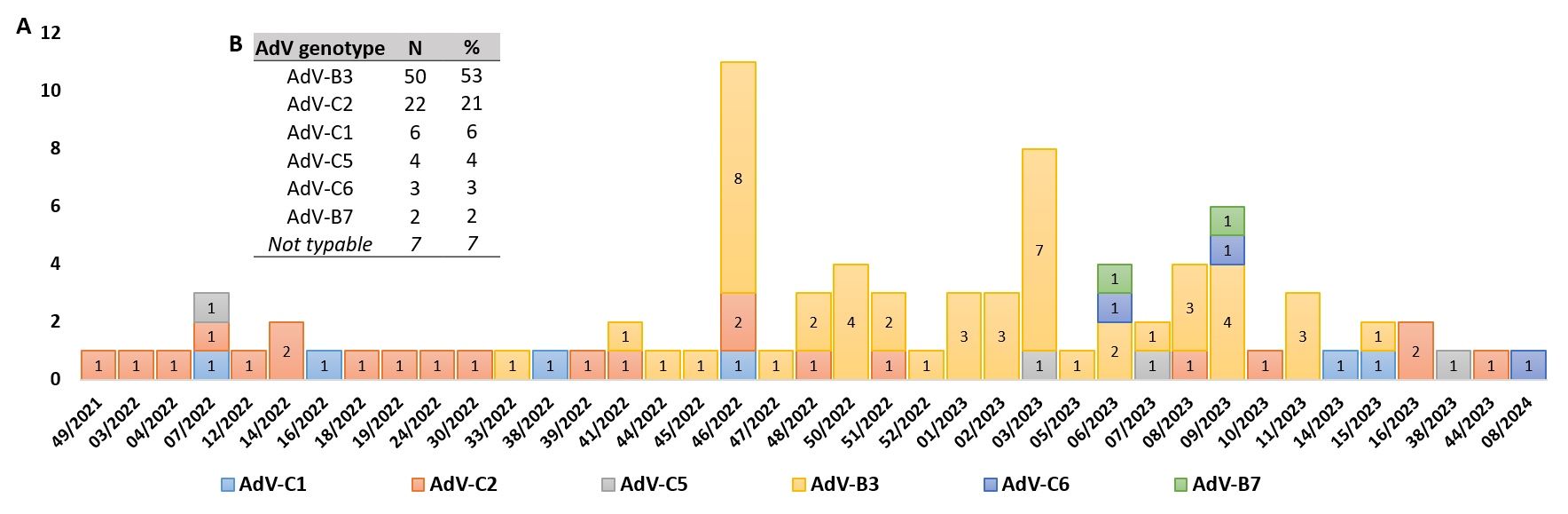

Supplement: Supplementary file 4 — Supplementary Material 4.Weekly distribution of SARS-CoV-2 molecular characterised-cases during the study period. (PNG 91.5 KB) [file 10096_2025_5353_MOESM4_ESM.png]
